# Editorial: Neurocardiology: the science of heart–brain interactions

**DOI:** 10.3389/fneur.2026.1921214

**Published:** 2026-07-21

**Authors:** Shubham Tomar, Karita Claudia Freitas Lidani, Ravinder Mallugari, Katherine Longardner, Bhavya Jaya Venugopal

**Affiliations:** 1 Division of Cardiovascular Medicine, Vanderbilt University Medical Center, Nashville, TN, United States; 2 Department of Neurosciences, University of California San Diego, La Jolla, CA, United States; 3 Department of Neurology, University of Kentucky, Lexington, KY, United States

**Keywords:** cardiovascular diseases, cerebrovascular imaging, cognitive decline, cognitive function, heart–brain axis

For decades, cardiology and neurology have largely evolved as separate disciplines, each focused-on understanding disease within the confines of a single organ system. The heart and brain “crosstalk” is a bidirectional structural and functional network linked through a dynamic exchange of autonomic, neurohumoral, inflammatory, vascular, and electrophysiological information to maintain homeostasis (1). Disruption of this network can initiate pathological processes in either organ, which subsequently propagate across systems, amplifying disease burden and contributing to adverse cardiovascular and neurological outcomes. The emerging field of neurocardiology seeks to understand these interactions and their implications for both research and clinical practice. The Research Topic, “*Neurocardiology: the science of heart–brain interactions*,” brings together studies in epidemiology, vascular biology, physiology, cognition, neuroimaging, genetics, and neuroscience. These collective works highlight that cardiovascular and neurological disorders often arise from intertwined biological pathways rather than isolated organ issues. The focus is shifting from describing associations to uncovering the mechanisms linking cardiac dysfunction, cerebrovascular injury, cognitive decline, and neurodegeneration. Key themes include vascular injury, cerebral microvascular disease, neuroinflammation, blood–brain barrier dysfunction, and hemodynamic measures like blood pressure profiles (Feng et al.; Genius et al.; Sun et al.; Kleven et al.; Bagheri et al.).

**Figure 1:**
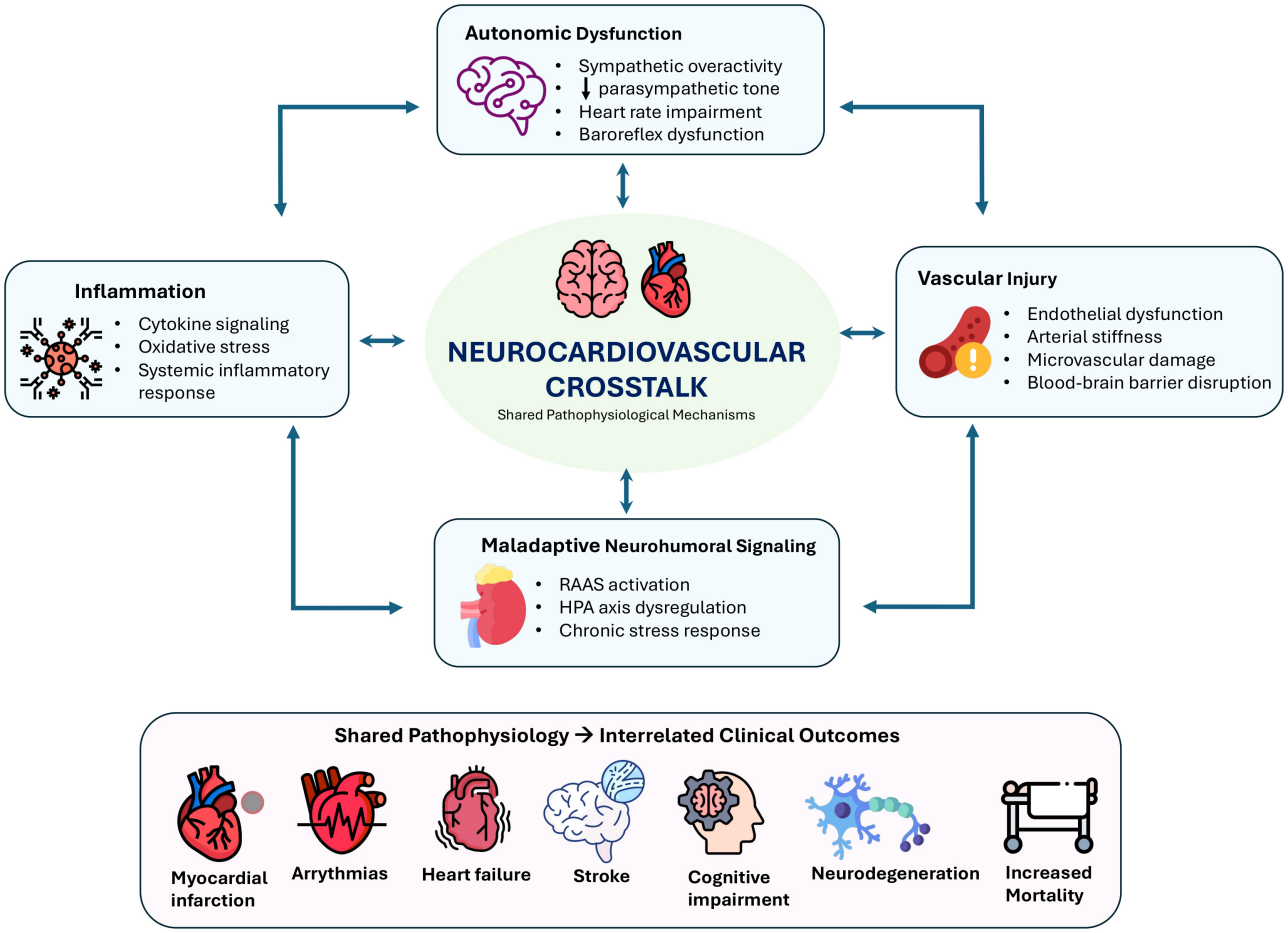
Shared biological pathways linking neurological and cardiovascular disorders. Autonomic dysfunction, chronic inflammation, vascular injury, and maladaptive neurohumoral signaling interact bidirectionally, creating a self-reinforcing cycle that contributes to neurocardiovascular disease progression and adverse clinical outcomes. HPA, Hypothalamic-Pituitary-Adrenal; RAAS, Renin-Angiotensin-Aldosterone System.

The concept of a bidirectional heart–brain continuum is supported by an expanding body of epidemiological and mechanistic evidence. Cardiovascular disease is associated with an increased risk of stroke, cognitive impairment, dementia, depression, and other neuropsychiatric disorders, while neurological and psychiatric conditions confer substantial cardiovascular risk (1). The influence of psychosocial factors on cardiovascular health provides a compelling example of this relationship. The INTERHEART study demonstrated that psychosocial stress accounts for nearly one-third of the population-attributable risk of myocardial infarction, an effect comparable to several traditional cardiovascular risk factors (2). Likewise, depression affects a substantial proportion of patients with heart failure and is associated with worse clinical outcomes (3). At the same time, cardiovascular disorders such as atrial fibrillation, myocardial infarction, and heart failure have consistently been linked to accelerated cognitive decline and increased dementia risk (1). Emerging evidence suggests that even subclinical cardiovascular injury may contribute to future neurological dysfunction, raising the possibility of common upstream mechanisms. Among these mechanisms, autonomic dysregulation occupies a central role. Reduced parasympathetic activity and heightened sympathetic drive contribute to endothelial dysfunction, arrhythmogenesis, vascular injury, and adverse cardiac remodeling while simultaneously influencing emotional regulation and cognitive performance (4). Inflammation represents another major interface. Chronic stress and cardiovascular disease promote the release of inflammatory mediators that contribute to atherosclerotic progression and vascular dysfunction. These same mediators can cross the blood–brain barrier, activate microglia, alter neurotransmitter metabolism, and promote neurodegenerative processes (5) (Figure 1).

Several studies featured in this Research Topic illustrate how neurocardiology is evolving from a field defined by clinical associations to one increasingly focused on biological mechanisms. A bibliometric analysis encompassing more than 3,800 publications demonstrated a marked acceleration in research activity over the past decade and highlighted a shift toward investigations of neuroinflammation, microglial activation, and blood–brain barrier dysfunction as potential mediators of vascular cognitive impairment (Sun et al.). This evolution reflects growing recognition that vascular pathology contributes to neurological dysfunction through mechanisms extending beyond overt stroke. Complementing these observations, one study demonstrated that genetic susceptibility to cerebral T2 white matter hyperintensities on MRI is associated with greater lesion burden and that hemodynamic factors, including blood pressure, partially mediate this relationship (Genius et al.). These findings underscore the interaction between inherited predisposition and modifiable cardiovascular risk factors in shaping cerebral vascular injury. Another contribution, based on data from the HUNT Study, demonstrated that elevated midlife cardiovascular risk predicts the development of mild cognitive impairment and dementia decades later (Kleven et al.). Similarly, a retrospective study examining carotid atherosclerotic plaque characteristics revealed significant associations between plaque burden and white matter hyperintensity severity (Feng et al.). Such findings reinforce the importance of cardiovascular health across the lifespan and suggest that neurological outcomes may be influenced long before the onset of clinical symptoms. Extending this concept to neurodegenerative disease, Bagheri et al. reported that neurogenic orthostatic hypotension in Parkinson's disease was associated with accelerated cognitive decline over longitudinal follow-up. By linking autonomic dysfunction and chronic blood pressure dysregulation to worsening cognitive trajectories, this study highlights a potential mechanism through which cardiovascular and neurological pathology interact (Bagheri et al.). Taken together, these findings provide further evidence that subclinical vascular disease may contribute to cumulative cerebral injury through mechanisms such as chronic hypoperfusion, endothelial dysfunction, and microvascular damage. Collectively, these studies advance an important conceptual shift. Rather than viewing cardiovascular disease and neurodegeneration as parallel processes that occasionally intersect, contemporary research increasingly recognizes them as interconnected outcomes arising from shared biological vulnerabilities.

Despite substantial progress, several critical questions remain unresolved. One of the most important challenges involves establishing the temporal sequence of events within the heart–brain continuum. Existing evidence supports both directions of influence between heart and brain. This apparent contradiction may stem from an overly simplistic view of causality. Rather than representing a linear pathway, the relationship between the heart and brain may be better understood as a self-reinforcing complex feedback loop. Cardiovascular dysfunction can impair cerebral perfusion, increase neuroinflammation, and accelerate neurodegenerative processes. In turn, neurological and psychiatric disorders can promote autonomic dysregulation, inflammation, maladaptive behaviors, and cardiovascular injury. Once established, this cycle may progressively amplify pathology in both organ systems. Another unresolved issue concerns the identification of biomarkers capable of detecting early neurocardiac disturbances before irreversible injury develops. Although advances in neuroimaging, cardiovascular phenotyping, and molecular biomarkers have improved risk stratification, critical gaps remain in defining the biomarkers that most accurately identify actionable disease and in defining the therapeutic windows during which intervention can meaningfully alter outcomes.

The future of neurocardiology will depend on how effectively we can integrate mechanistic discovery with clinical implementation. A major priority should be the identification of subclinical cardiovascular and neurological injury through multimodal approaches incorporating clinical, imaging, physiological assessment, and biomarker profiling. Earlier recognition of vulnerable individuals may provide opportunities to intervene before overt disease develops. Integrated models of care also warrant consideration. Routine cognitive assessment in selected cardiovascular populations and cardiovascular risk evaluation among patients with neurological disorders may facilitate earlier recognition of shared pathology. Multidisciplinary “Heart–Brain Teams” could provide a practical framework for managing patients whose conditions transcend traditional specialty boundaries. Emerging therapeutic strategies targeting the biological interfaces between the heart and brain represent another promising avenue. Neuromodulatory approaches, including vagus nerve stimulation, as well as therapies directed toward chronic inflammation and immune dysregulation, may offer opportunities to disrupt the pathological feedback loops linking cardiovascular and neurological disease (6). However, translating these advances into evidence-based clinical practice will require a better understanding of which patients are most likely to benefit, the mechanisms underlying treatment responsiveness, and whether benefits persist over time. Ultimately, future longitudinal studies integrating cardiovascular, neurological, imaging, biomarker, and behavioral data will be essential for clarifying disease trajectories and identifying actionable intervention points. Determining whether early treatment of subclinical myocardial injury, autonomic dysfunction, or vascular disease can prevent downstream neurodegeneration remains one of the most important unanswered questions in the field.

The growing body of evidence supporting bidirectional heart–brain interactions challenges traditional organ-specific models of diseases. The studies featured in this Research Topic reinforce the view that cardiovascular and neurological disorders frequently arise from shared biological pathways involving autonomic dysfunction, inflammation, vascular injury, and maladaptive neurohumoral signaling. Rather than asking whether disease originates in the heart or the brain, future research should focus on understanding how pathology propagates between these interconnected systems. Embracing this systems-based perspective represents not only a conceptual advance but also a necessary step toward developing integrated strategies for prevention, diagnosis, and treatment. As neurocardiology continues to mature, interrupting the self-reinforcing cycle linking cardiovascular and neurological disease may become one of the most important opportunities for improving long-term health outcomes.
